# Epalrestat, an aldose reductase inhibitor, in diabetic neuropathy: An Indian perspective

**DOI:** 10.4103/0972-2327.44558

**Published:** 2008

**Authors:** S. R. Sharma, Nalini Sharma

**Affiliations:** Department of Neurology, King George Medical University, Lucknow, India

**Keywords:** Diabetes, peripheral neuropathy, aldolase reductase inhibitor

## Abstract

**Background:**

A number of diabetic patients with diabetic neuropathy, in India, were treated with epalrestat, an aldose reductase inhibitor. In this study, more than 2000 patients with diabetic neuropathy, who were treated with epalrestat for 3-12 months, were analyzed to assess the efficacy and the adverse reactions of the drug.

**Method:**

We analyzed the subjective symptoms (spontaneous pain, numbness, coldness and hypoesthesia) and the nerve function tests (motor nerve conduction velocity, sensory nerve conduction velocity and vibration threshold).

**Result:**

The improvement rate of the subjective symptoms was 75% (slightly improved or better) and that of the nerve function tests 36%. Adverse drug reactions were encountered in 52 (2.5%) of the 2190 patients, none of which was severe.

**Conclusion:**

Although data are limited, it is strongly suggested that epalrestat is a highly effective and safe agent for the treatment of diabetic neuropathy.

## Introduction

Epalrestat is a carboxylic acid derivative that inhibits aldose reductase, a rate-limiting enzyme of the polyol pathways.[1–3] In hyperglycemia, epalrestat, an uncompetitive aldose reductase inhibitor, significantly reduces intracellular sorbitol accumulation, which has been related in the pathogenesis of late-onset diabetic complication like neuropathy, retinopathy and nephropathy.[2–3]

It has been confirmed in diabetic animal experiments that epalrestat improved nerve-conduction velocity, and this was accompanied by an improvement of sorbitol levels and Na+/K+- AT Pase activity in the nerve. An improvement in the morphological abnormalities of the nerves was also observed in epalrestat treated diabetics rats.[5] Then, a placebo-controlled double-blind clinical study of epalrestat was carried out and the effectiveness of the drug against diabetic neuropathy documented.[10]

We conducted a clinical study in diabetic neuropathy at our centre, to confirm the efficacy and safety of epalrestat for 12 to 24 weeks. In this study, we analyze the data of subjective symptoms and nerve conduction study on 2190 cases with diabetic neuropathy, treated with epalrestat.

## Methods

Adults aged ≥20 years were diagnosed as mild diabetic peripheral neuropathy based on subjective symptoms, no foot ulcer, and neurological dysfunctions (at least two parameters: MNCV [indispensable] and vibration perception threshold (VPT) or Achilles tendon reflex etc.). Patients were enrolled for the study if they had a motor nerve conduction velocity (MNCV) ≥40 m/s (seemingly reversible) and stable glycemic control (HbA_1c_ [A1C] ≤9%, with ±0.5% variation in the previous three months). Subjects were excluded if their primary cause of neurologic disorder was not diabetes (alcoholic neuropathy, carpal tunnel syndrome, sequelae of cerebrovascular disease etc.), if they had arteriosclerosis obliterans (ankle brachial pressure index of ≤0.8) or severe hepatic or renal disorder, if they were participating in other interventional studies, or if they were receiving other experimental medications for diabetic neuropathy, prostaglandin E_1_ preparations or any other medication that affects symptoms of diabetic neuropathy.

Sensory symptoms were usually presenting complaints and were either positive (paresthesias/dysesthesias) or negative (loss or impairment of different modalities). Neuropathic pain had deep, bursting, drawing character and was associated with jabbing or shooting pains, which typically increased with rest. Motor symptoms were either negative (paralysis of voluntary muscles) or positive (fasciculations, myokimia, muscle cramps). Autonomic dysfunction led to orthostatic fainting spells, diahrroea, constipation, impotence, sphincter disturbances or sweating disturbances. Anorexia, early satiety and nausea were symptoms of gastroparesis.

### Characteristics of patients

The dosage of epalrestat was 150 mg/day. One tablet (50mg) was orally administered three times daily before each meal. The number of patients who received epalrestat 150 mg daily for more than three months accounted for 84.2% (n = 1844) and for more than six months 49.1% (n =1075).

As to the background of the 2190 patients, the gender ratio was close to 1. The average age was 63 years. Epalrestat was administered in patients primarily with diabetes mellitus for more than 10 years, with an average duration of 11.3 ± 0.2 years (mean ± SEM).

The average duration of diabetic neuropathy was 3.2±0.1 years. The drug was administered in most of the patients with mild to moderate severity of diabetic neuropathy, as shown in Table 1. Among 2190 cases, about 90% (n = 1971) had non-insulin-dependent diabetes mellitus (NIDDM), whereas about 10 % (n= 219) had insulin-dependent diabetes mellitus (IDDM). Only 20 % (n = 438) was under dietary therapy; others were under medical treatment with oral hypoglycemic agents (n = 896), insulin injection (n = 836) or its combined therapies (n = 20). As for blood glucose control, the fasting blood glucose level was 172 ± 8 mg/dL and the average HbA1c level was 8.4 ± 0.1%. Of these, 55 withdrew after two months; the reasons for withdrawal were change in hospital,[12] complications in comorbid illnesses,[7] amelioration of symptoms (two epalrestat), adverse effects (AEs) (20 epalrestat), and deterioration in symptoms.[14]

**Table 1:** Characterstics of patients (N = 2190)

Gender	
Male	1120
Female	1070
Age (years)	
<=29	15
30-59	805
=>60	1370
Mean ± SEM	60.1±0.2
Duration of diabetes (years)	
<3	274
3.1 - 5.0	342
5.1 - 10.0	578
>10.1	996
Mean ± SEM	11.3± 0.2
Status of Diabetic neuropathy	
Duration of neuropathy (years)	
<1	270
1.1-3	246
3.1-5	590
>5.1	984
Mean ±SEM	3.2± 0.1
Severity of neuropathy	
Mild	1011
Moderate	905
Severe	274
Total number	2190

Study end points and measures of outcome;- study visit occurred at six monthly intervals

### Statistical analysis

Assuming that the population SD of median MNCV is 60 m/s, statistical methods used included *x*[2] tests for nominal scale, Mann-Whitney U tests for ordered categorical scale, two-sample t tests for comparison of mean values beween groups, paired t tests for comparison of mean values.

## Results

Effects of epalrestat on subjective symptoms and nerve function tests

**Figure 1:**
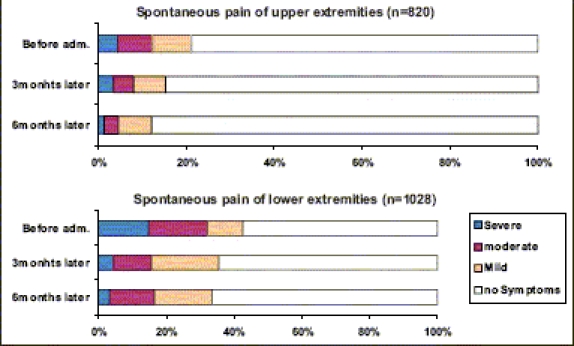
Improvement of symptoms-painful neuropathy

**Figure 2:**
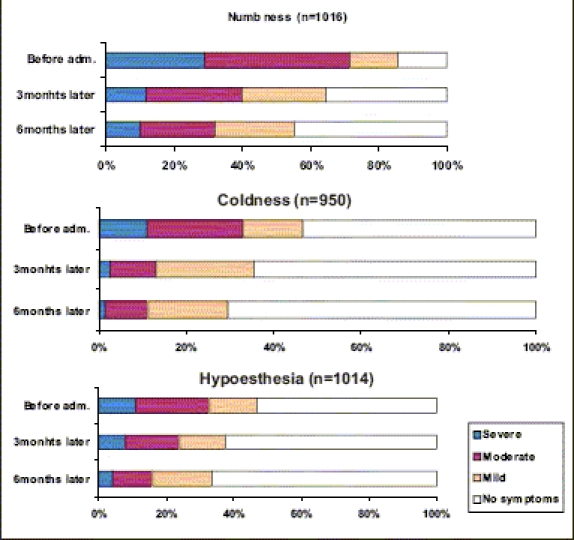
Improvement of symptoms: sensory disorder Wilcoxon's U-test ***: *P* < 0.001 vs. before adm.

Figure 1 shows the efficacy of epalrestat on spontaneous pain of upper or lower extremities after three months or six months of administration. Significant improvement of spontaneous pain was observed in both upper and lower extremities, after the administration of epalrestat for three months, and its effect became greater after administration for six months. Similar to the effect on spontaneous pain, epalrestat significantly improved the sensory disorders (numbness, coldness and hypoesthesia), after administration for three months, and the effect became greater after six months. [Figure 2] Significant improvement was observed especially in patients with moderate symptoms. On the other hand, with regard to autonomic nerve dysfunction, the drug markedly proved effective on orthostatic dizziness and constipation, but it was not effective on diarrhea.

**Figure 3:**
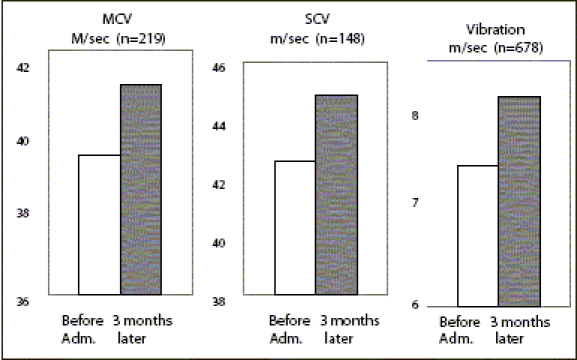
Nerve function tests Mean +SEM Paired test *: *P* < 0.05 ***: *P* < 0.001 vs. Before adm. N= 52 (2.5%)

*Nerve Function tests:* The primary end point was change from the baseline to the study end in MNCV. Figure 3 shows the results on nerve function test after administration of epalrestat for three months. The drug significantly increased peroneal motor nerve-conduction velocity and sural sensory nerve-conduction velocity. Similarly, VPT was measured on the medial malleolus in the lower extremities, using a 128-Hz tuning fork and measuring the number of seconds, until the patient could no longer feel the vibrations after the tuning fork was placed on the medial malleolus.

Vibration sensitivity, measured with a C-128 tuning fork, markedly increased after administration of epalrestat for three months. The same nerve function tests were conducted after six months of administration of the drug, and significant improvements were observed again in peroneal motor nerve-conduction velocity and vibration sensitivity [Tables 2 and 3].

**Table 2:** Change in nerve function over time

Variables MNCV (m/s)	n	Baseline	6 Months	
			
			Δ	P*
Epalrestat	219	51.96 ± 4.49	+0.29 ± 3.11	0.214
P‡		0.002	0.013	
VPT (s)				
Epalrestat	678	8.01 ± 3.43	+0.57 ± 3.21	0.110
P‡		0.147	0.095	

Data are means ± SD, unless otherwise indicated. Δ, change vs. baseline.

* P values were calculated using paired t test and

‡ two-sample t test, and VPT was measured on the medial malleolus in the low extremities, using a 128-Hz tuning fork by measuring the number of seconds until the patient could no longer feel the vibrations after the tuning fork was placed on the medial malleolus.

**Table 3:** Baseline electrophysiologic and vibration perception threshold data

Neurophysiologic measures	Values
SNCV median, m/sec	55.3 ± 0.8
SNCV sural, m/sec	43.3 ± 0.8
MNCV peroneal, m/sec	40.5 ± 0.8
SNAP-amplitude median, μV	17.9 ± 1.9
SNAP–amplitude sural, μV	7.23 ± 0.82
M–amplitude peroneal, mV	3.88 ± 0.35
Vibration perception threshold toe, μm	13.2 ± 2.1

NCV = nerve conduction velocity; SNCV = sensory nerve conduction velocity; MNCV = motor nerve conduction velocity; SNAP = sensory nerve action potential.

### Assessment of efficacy by attending physicians

*Subjective symptoms:* Reductions in the symptom levels of ≥50% or <50% was considered an improvement or semi-improvement; no reduction was considered unchanged, and an increase was considered to be aggravation.

Figure 2 shows the clinical improvements of the subjective symptoms and the nerve test function tests. The improvement ratio of the subjective symptoms was 75% and the corresponding rating of the nerve function tests was 36%, showing greater efficacy in the improvement of subjective symptoms, relative to nerve function tests. The combined overall improvement rating was judged to be 76% [Table 4].

**Table 4:** Judgment of efficacy by attending physicians: Clinical improvement

Improvement Rating (%)	Remarkably Improved	slightly Improved	Improved	unchanged	aggravated	Total no.	(≥ Slightly improved)
Subjective	9.90%	32.02	33.10%	24.21%	0.75%	2169	75
Symptoms	(220)	(700)		(730)	(510)		(9)
Nerve function	2.34%	14.64%	19.48%	59.76%	3.76%	561	
Test (561)	(14)	(83)	(110)	(320)	(22)		36
Overall improved	8.05%	32.75%	35.36%	23.02%	nil	2180	76
Rating (2180)	(18)	(720)		(770)	(510)		

*Adverse Reactions by Epalrestat:* The details of adverse reactions in our present study in which epalrestat has been or was being administered in 2190 patients are summarized in Table 5. Adverse reactions were reported in 52 cases (2.5%). The most frequent adverse reaction was hepatic dysfunction, and all the cases with hepatic dysfunction had elevation of Serum glutamic oxaloacetic acid transferase (SGOT) and Serum glutamic pyruvate transferase (SGPT). None of these adverse reactions was serious.

**Table 5:** Adverse reactions: occurred in 52 out of 2190 cases (2.5%)

Symptoms	Number of incidences	%
Hepatic dysfunction	19	36.5%
Nausea / Vomiting	5	9.6%
Gastric discomfort	4	7.6%
Eruption	1	1.9%
Exacerbation of renal function	1	1.9%
Exacerbation of systemic numbness	1	1.9%
Edema	1	1.9%
Diarrhea	2	3.8%
Others	18	35%
Total	52	100

From the analysis made by attending physicians, it seems that safety was not particularly a problem in 98% patients. The utility or the comprehensive assessment of all data was as high as 76%.

## Discussion

Of the patients with diabetic neuropathy, 60–70% will develop serious complications that will culminate in the amputation of an appendage.[12] It is, therefore, of paramount importance to treat diabetic neuropathies appropriately.

Generally, median MNCV decreases with time in patients with diabetic neuropathy. Partanen *et al.*[13] reported a significant decrease of 2.9 m/s in the median MNCV over a 10-year period in patients with type 2 diabetes, which converts to a reduction over three years of 0.87 m/s. Other Aldose Reductase Inhibitors (ARIs), including fidarestat, ranirestat and zenarestat, have also been reported to improve motor nerve conduction velocity (MNCV) and motor nerve f wave latency (MFWL). Although a 52-week study of fidarestat did not show a significant effect on median MNCV, the median nerve F-wave conduction velocity and minimal latency were improved significantly.[14] Moreover, in another 52-week study,[15] zenarestat considerably improved peroneal MNCV. Similarly, ranirestat was shown to improve sensory nerve conduction by ≥1 m/s over 12 weeks[16] and peroneal MNCV after 60 weeks of treatment.[17]

Short-term treatment with fidarestat (28 weeks) remarkably improved performance in arm and leg VPT, an indicator of sensory nerve disturbance.[18] Raniresat also improved VPT on the first toe following 60 weeks of treatment.[17] In our study, VPT deteriorated over time in the control group but did not change significantly in the epalrestat group, suggesting that epalrestat is effective in preserving the sensory function. This is further supported by the improvement in the subjective symptoms (numbness of limbs, sensory abnormality, and cramping) with epalrestat therapy, although such improvements are difficult to evaluate in an open-label study. However, because the nerve function inspection by the medical technologist and the assessment of the electromyogram by the specialized physician were carried out under masked conditions, it is thought that bias has been minimized.

In performing a stratified analysis using median MNCV as an index, epalrestat was most effective in subjects with good glycemic control. Hyperglycemia-induced hyperactivity of polyol pathway links to the augmentation of metabolic disorders like glycation, oxidative stress, and others, contributing to the deterioration of diabetic neuropathy. However, these disorders are not completely caused from the hyperactivity of polyol pathway.[6–9] Therefore, our data suggest that good glycemic control may be important to keep the better effect of aldose reductase inhibitor (ARI) treatment.

Adverse effects attributed to epalrestat were previously reported in 3.0% of the subjects in a 12-week study[10] and 129 of 5,249 subjects (2.5%) in a 3 to 12-month multicenter study.[11] The higher incidence (8.8%) of AEs in this study may be due to the longer duration of the study. It should be, however noted that no particularly severe events were observed, thus confirming the safety of epalrestat for long-term administration.

When administered to 2190 patients suffering from diabetic neuropathy for more than three months, epalrestat exerted an improvement in the subjective symptoms and the nerve functions test. From the analysis of data, it appears that the longer the duration of administration was, the greater the improvement rate became. Adverse drug reactions were encountered in 52 cases (2.5 %) out of 2190, but none of them was severe. From these findings, it is clear that epalrestat is a highly effective and safe drug against diabetic neuropathy. Effects are particularly evident in patients with good glycemic control.

It is important for us to collect much more data in an increased number of patients over longer period of time for evaluating the efficacy and safety of epalrestat.
